# Integrated genomic and phenotypic analysis of an endophytic bacterium reveals biocontrol and plant growth-promoting mechanisms

**DOI:** 10.1016/j.isci.2026.116210

**Published:** 2026-06-14

**Authors:** Rongbo Sa, Zhijie Cao, Qin Luo, Xinru Liu, Shuxin Ma, Guoqing Zhang, Junli Zhang, Yachao Zhang, Meimei Song

**Affiliations:** 1 School of Life Sciences, Shandong First Medical University & Shandong Academy of Medical Sciences, Taian 271000, China

**Keywords:** applied microbiology, plant biology, interaction of plants with organisms, plant physiology

## Abstract

Root rot threatens *Salvia miltiorrhiza* yield and quality. We isolated an endophytic bacterium, *Paenibacillus polymyxa* DS-S6, from healthy plant stems that showed strong antifungal activity against *Fusarium solani* and other phytopathogens. The strain exhibited multiple plant growth-promoting traits, including phosphate solubilization, siderophore production, and secretion of indole-3-acetic acid-like compounds. Pot experiments confirmed that DS-S6 significantly promoted seedling growth and suppressed *Fusarium* root rot. The complete 5.84 Mb genome revealed 18 biosynthetic gene clusters, with five showing 100% similarity to known antifungal lipopeptide clusters. LC-MS confirmed production of fusaricidins and paenibacillin. GFP-tagged DS-S6 successfully colonized rhizosphere and internal tissues for at least 50 days. This integrated analysis positions DS-S6 as a promising multi-functional candidate for sustainable biocontrol and growth promotion in *S*. *miltiorrhiza* cultivation.

## Highlights

• Complete genome of an endophytic bacterium reveals 18 BGCs
• LC-MS confirms production of antifungal fusaricidins and paenibacillin
• The bacterium colonizes *S*. *miltiorrhiza* tissues for at least 50 days
• The bacterium suppresses Fusarium root rot and promotes plant growth

## Graphical abstract

**Figure:**
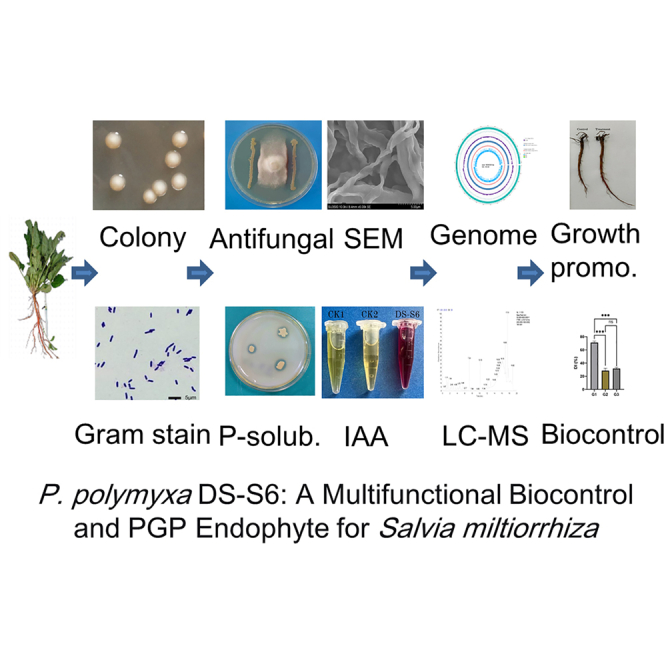

## Introduction

*Salvia miltiorrhiza*, a cornerstone of traditional Chinese medicine, faces a significant threat from root rot disease, which severely compromises both its yield and quality. The expanding cultivation of *S*. *miltiorrhiza* has been paralleled by an increase in the incidence of root rot, with severe cases leading to yield losses exceeding 40%.^1^^,^^2^ Pathogenic fungi, primarily *Fusarium solani*, *Fusarium proliferatum*, and *Fusarium oxysporum*, have been identified as the main causative agents.^3^ While current management relies heavily on chemical fungicides, their suboptimal efficacy and the associated risks of pesticide residues and environmental contamination underscore the urgent need for sustainable alternatives.^4^^,^^5^

Biological control, utilizing beneficial microorganisms, has emerged as a promising strategy. Endophytic microorganisms, which reside within plant tissues without causing disease, are of particular interest due to their direct access to pathogens and potential for enhancing plant growth.^6^ Previous research on *S*. *miltiorrhiza* endophytes has predominantly focused on fungi, exploring their diversity and antagonistic potential.^3^^,^^7^ In contrast, the potential of endophytic bacteria as biocontrol agents against *S*. *miltiorrhiza* root rot remains relatively unexplored.

Among beneficial bacteria, *Paenibacillus polymyxa* stands out as a plant growth-promoting rhizobacterium with a formidable reputation for broad-spectrum antimicrobial activity and an excellent safety profile.^8^ This bacterium employs multiple mechanisms to benefit plants, including nitrogen fixation, phosphate solubilization, siderophore production, and phytohormone secretion.^9^ For instance, *P*. *polymyxa* Sx3 has been shown to promote rice growth through nitrogen fixation, phosphate solubilization, and indole-3-acetic acid (IAA) production.^10^ Its biocontrol prowess is attributed to niche competition, induction of systemic resistance in plants, and the production of an arsenal of antimicrobial compounds, such as polypeptide antibiotics (e.g., polymyxin, fusaricidin, etc.), proteins, and other secondary metabolites.^11^^,^^12^

Traditional microbiological methods often fall short of fully elucidating the genetic basis of these complex traits. The advent of whole-genome sequencing has revolutionized our ability to decipher the functional potential of microorganisms.^13^ For *P*. *polymyxa*, genomics has enabled the systematic identification of gene clusters responsible for antibiotic synthesis and plant growth promotion, directly linking genomic features to beneficial phenotypes, as demonstrated in the model strain E681.^14^^,^^15^

In our previous work, we isolated 72 bacterial strains from healthy *S*. *miltiorrhiza* tissues, among which strain DS-S6, originating from the stem, exhibited the most potent antagonism against *F*. *solani*.^4^ Preliminary identification classified it as *P*. *polymyxa*. To comprehensively unravel the genetic determinants of its biocontrol and growth-promoting capabilities, we herein present a complete genome sequence analysis of DS-S6. This study integrates genomic mining with phenotypic validation to (1) identify genes and gene clusters involved in antimicrobial compound synthesis, plant growth promotion, and colonization; (2) experimentally verify the production of key predicted metabolites; and (3) confirm its endophytic colonization ability and plant growth-promoting effects in *S*. *miltiorrhiza*. Our work provides a foundational understanding of DS-S6’s mode of action, facilitating its future development as an effective biocontrol agent.

## Results

### Morphological and phylogenetic identification of strain DS-S6

**Figure 1:**
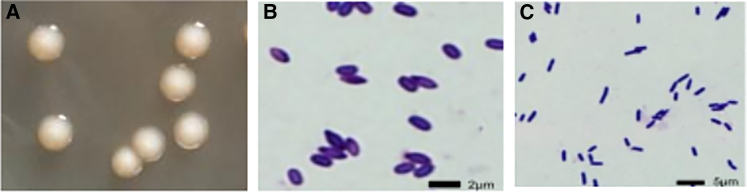
Morphological characteristics of strain DS-S6 (A) Colony morphology of strain DS-S6 on LB agar, (B) Endospore staining result of DS-S6 cells. Scale bars, 2 μm. (C) Gram staining result of DS-S6 cells. Scale bars, 5 μm.

Strain DS-S6 formed creamy-white, opaque, convex colonies with regular edges and a sticky consistency on LB agar (Figure 1A). Microscopic examination revealed short rod-shaped cells capable of forming capsules and endospores (Figure 1B), and the strain was confirmed to be Gram-positive (Figure 1C).

**Figure 2:**
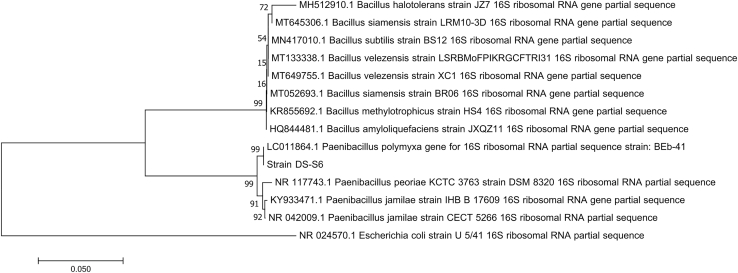
The phylogenetic tree based on 16 S rDNA sequence

Physiological and biochemical profiling indicated positive results for citrate utilization, gelatin liquefaction, amylohydrolysis, nitrate reduction, catalase, and V-P reaction (Table 1). Phylogenetic analysis of the 1,445-bp 16 S rDNA sequence revealed a 99.65% similarity to *P*. *polymyxa* strain LC011864, forming a distinct clade with this type strain (Figure 2). Collectively, these results unequivocally identify strain DS-S6 as *P*. *polymyxa*.

**Table 1:** Physiological and biochemical properties of strain DS-S6

Property	Result	Property	Result
Citrate utilization	+	Amylohydrolysis	+
Gelatin liquefaction	+	Catalase	+
Oxidase	–	Casein hydrolysis	+
Succinate utilization	–	V-P reaction	+
Glucose	+	Methyl red test	–
Nitrate reduction	+	H_2_S production	–
Isinglass hydrolysis	+	Tolerance to NaCl	<7%

Note, “+” positive; “–” negative.

### Assessment of antifungal activity

**Figure 3:**
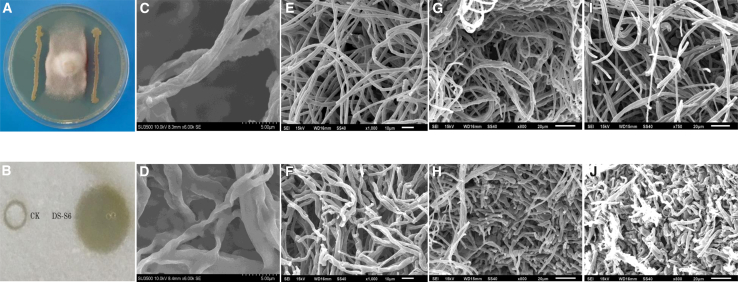
Antifungal activity of strain DS-S6 against *F*. *solani* and ultrastructural damage to fungal hyphae by SEM observation (A) Dual culture assay, (B) conidial germination assay, (C) *F*. *solani* normal hyphae, (D) *F*. *solani* suppressed hyphae, (E) *R*. *solani* normal hyphae, (F) *R*. *solani* suppressed hyphae, (G) *A*. *alternata* normal hyphae, (H) *A*. *alternate* suppressed hyphae, (I) *F*. *oxysporum* normal hyphae, (J) *F*. *oxysporum* suppressed hyphae. Scale bars, 5 μm (C and D), 10 μm (E and F) and 20 μm (G–J).

Strain DS-S6 displayed potent antifungal activity against *F*. *solani in vitro*. In dual culture assays, a distinct inhibition zone was observed between DS-S6 and the fungal colony, indicating direct suppression of mycelial growth (Figure 3A). The conidial germination plate assay showed that the addition of cell-free culture supernatant of DS-S6 inhibited the germination of *F*. *solani* conidia into hyphae, resulting in a distinct inhibition zone. In contrast, conidia in the control group germinated normally into hyphae, with no inhibition zone observed (Figure 3B).

Dual culture assay demonstrated the potent antifungal activity of DS-S6 against several phytopathogenic fungi. SEM further revealed severe structural damage to fungal hyphae following treatment with DS-S6. Untreated hyphae of *F*. *solani*, *R*. *solani*, *A*. *alternata*, and *F*. *oxysporum* exhibited normal, smooth, and turgid morphology (Figures 3C–3E, 3G, and 3I). In contrast, hyphae exposed to DS-S6 showed severe deformities, including excessive twisting, surface roughness, dehydration, shrinkage, abnormal swelling, and extensive fragmentation (Figures 3D–3F, 3H, and 3J), indicating a strong lytic effect.

### Plant growth-promoting traits

Strain DS-S6 exhibited multiple PGP characteristics *in vitro*, as assessed by standard qualitative screening assays.

#### Phosphate solubilization

**Figure 4:**
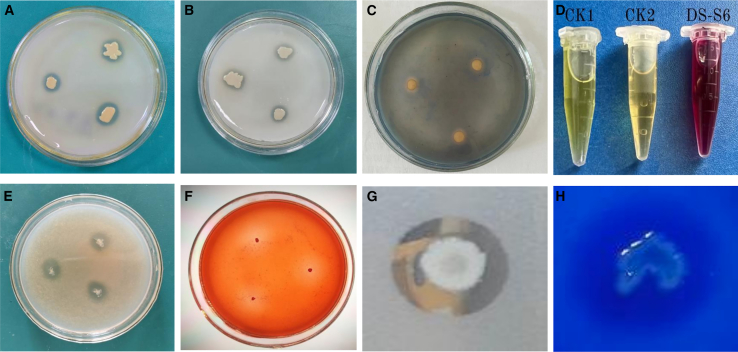
Biological characteristics involved in PGP activity of strain DS-S6 (A) Inorganic medium, (B) organic medium, (C) siderophore production, (D) IAA-like compounds secretion, (E) protease secretion, (F) cellulose secretion, (G) chitinase secretion, and (H) β-1,3-glucanase secretion.

DS-S6 formed clear solubilization halos on both inorganic (NBRIP) and organic (Pikovskaya) phosphorus agar plates, indicating positive activity for both forms of phosphate solubilization (Figures 4A and 4B).

#### Siderophore production

A distinct orange halo developed around DS-S6 colonies on CAS agar, confirming the ability to produce siderophores (Figure 4C).

#### IAA-like compound secretion

The Salkowski assay yielded an orange-red color in the culture supernatant of DS-S6 grown in LB broth supplemented with L-tryptophan, while both negative controls (CK1, sterile medium + tryptophan; CK2, DS-S6 without tryptophan) remained colorless. This result indicates the production of IAA-like compounds (Figure 4D).

#### Extracellular hydrolytic enzyme production

DS-S6 also demonstrated the capacity to secrete multiple hydrolytic enzymes, as evidenced by clear hydrolysis zones on agar plates supplemented with specific substrates: casein agar (protease), CMC-Na (cellulase), colloidal chitin (chitinase), and β-1,3-glucan agar (β-1,3-glucanase) (Figures 4E–4H).

### Plant growth-promotion in pot experiments

**Figure 5:**
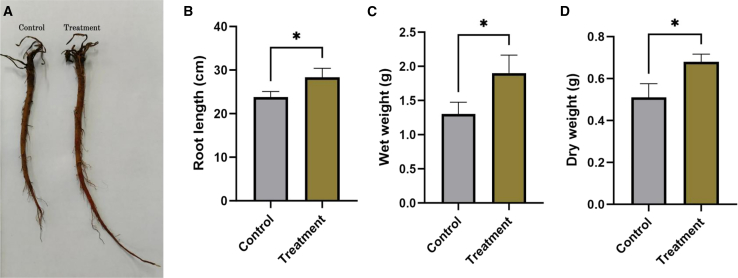
DS-S6 promotes root growth and biomass accumulation in *S*. *miltiorrhiza* under non-stressed conditions (A) Representative root systems of control and treatment group plants at 90 days after transplantation, (B–D) quantitative analysis of (B) root length, (C) root wet weight, and (D) root dry weight. Data are means ± SD (*n* = 10). Asterisks indicate significant differences control and treatment (∗*p* < 0.05, ∗∗*p* < 0.01, ∗∗∗*p* < 0.001; Student’s *t* test).

Inoculation with DS-S6 significantly enhanced the growth of *S*. *miltiorrhiza* seedlings under non-stressed conditions. Compared to the control group, treatment group exhibited longer and more branched roots, as shown in Figure 5A. Quantitative analysis revealed significant increases in average root length, wet weight, and dry weight (Figures 5B–5D). These results are summarized in Figure 5 and validate the plant growth-promoting potential of DS-S6 *in vivo*.

### DS-S6 suppresses *Fusarium* root rot and restores growth in *S*. *miltiorrhiza*

**Figure 6:**
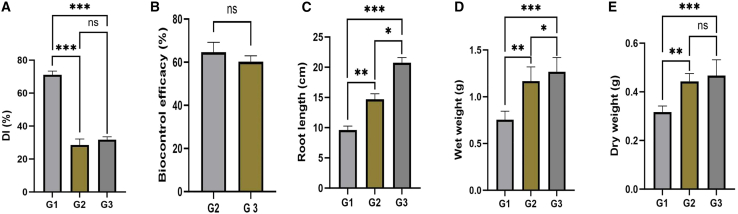
Biocontrol efficacy of DS-S6 against *Fusarium* root rot and growth recovery in *S*. *miltiorrhiza* (A) DI (%), (B) biocontrol efficacy, (C) root length, (D) wet weight, and (E) dry weight. Data are means ± SD (*n* = 10). Asterisks indicate significant differences control and treatment (∗*p* < 0.05, ∗∗*p* < 0.01, ∗∗∗*p* < 0.001; Student’s *t* test).

The final DI revealed that both DS-S6 and carbendazim significantly reduced *Fusarium*-induced root rot compared to the pathogen control (G1). The DI in G1 was 71.2%, whereas it decreased to 28.5% in G2 and 30.8% in G3 (Figure 6A), indicating substantial disease suppression by both treatments. Notably, the biocontrol efficacy of DS-S6 (63.7%) was comparable to that of carbendazim (68.4%), with no significant difference between the two (*p* > 0.05; Figure 6B), suggesting that DS-S6 is as effective as the chemical fungicide in controlling the disease.

In addition to disease suppression, DS-S6 significantly alleviated the growth inhibition caused by *F*. *solani*. Root length in G1 was only 9.5 cm, but increased to 14.5 cm in G2 and 20.5 cm in G3 (Figure 6C). Similarly, root fresh weight rose from 0.75 g in G1 to 1.15 g in G2 and 1.25 g in G3 (Figure 6D). Dry weight also showed a consistent trend, increasing from 0.30 g in G1 to 0.45 g in G2 and 0.48 g in G3 (Figure 6E). While G3 exhibited slightly higher root length and fresh weight than G2, the difference in dry weight between G2 and G3 was not statistically significant (*p* > 0.05), indicating that DS-S6 effectively restored plant biomass accumulation to a level comparable to that achieved by chemical treatment. These results demonstrate that DS-S6 not only suppresses *Fusarium* root rot but also mitigates the associated growth suppression, providing comprehensive protection to *S*. *miltiorrhiza* under pathogen stress.

### Biological functionality of the GFP-labeled strain

The constructed GFP-labeled strain DS-S6-GFP exhibited bright green fluorescence under blue light with an excitation wavelength of 480 nm (Figure S1). To ensure that GFP labeling did not impair key biocontrol traits, we compared the growth kinetics and antifungal activity of DS-S6-GFP with the wild-type strain. Both strains exhibited nearly identical growth profiles in LB broth over 36 h (Figure S2). Similarly, no significant difference was detected in the inhibition zone diameter against *F*. *solani* in dual culture assays (Figure S3). These results indicate that GFP labeling did not adversely affect the core biological functions of DS-S6 relevant to its plant-beneficial activities.

### General genomic features

**Figure 7:**
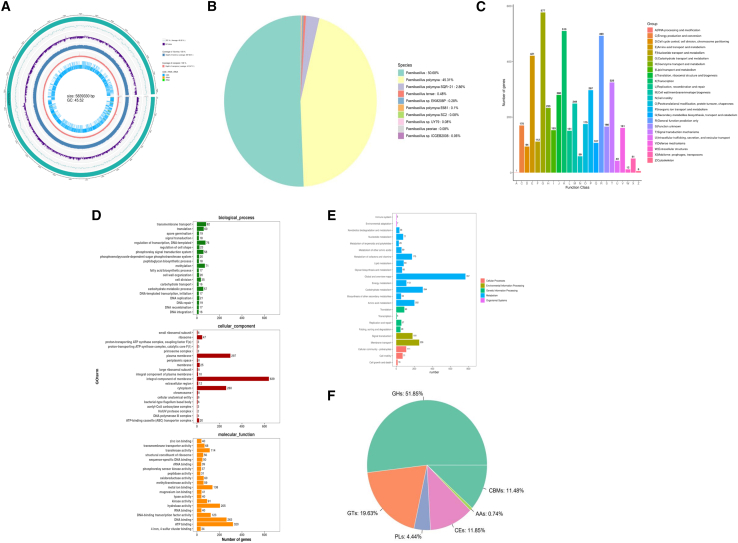
Analysis of genome structure and metabolic pathway of strain DS-S6 (A) Genome map of strain DS-S6, (B) NR annotation of strain DS-S6 genome, (C) COG annotation of strain DS-S6 genome, (D) GO annotation of strain DS-S6 genome, (E) KEGG pathway annotation of strain DS-S6 genome, and (F) gene count distributions of CAZy families.

The complete genome of *P*. *polymyxa* DS-S6 is a single circular chromosome of 5,839,330 bp with a G + C content of 45.52% (Figure 7A; Table 2). Genome annotation predicted 5,064 protein-CDS, 109 tRNAs, one tmRNA, 42 rRNAs (14 each of 5 S, 16 S, and 23 S), foour CRISPR arrays, and seven genomic islands. The raw data obtained from the whole-genome sequencing of strain DS-S6 was uploaded to NCBI (https://www.ncbi.nlm.nih.gov) and assigned the accession number PRJNA1301861. Based on the results of functional annotation, out of the 5,064 identified CDS, 5,048, 4,090, 2,850, and 2,945 were annotated into Non-Redundant Protein Database (NR), Cluster of Orthologous Groups of proteins (COG), Gene Ontology (GO), and Kyoto Encyclopedia of Genes and Genomes (KEGG) pathway categories, respectively.

**Table 2:** General features of the genome of the strain DS-S6

Features	Genome
Genome size (bp)	5,839,330
Gene number	5,272
Gene total length	5,108,547
G + C content (%)	45.52
CDS	5,064
tRNA gene	109
23 S rRNA gene	14
16 S rRNA gene	14
5 S rRNA gene	14
tmRNA	1
CRISPR-Cas	4
Genomic island	7
Genome accession	GenBank: PRJNA1301861

### Functional genomic annotation

#### NR functional annotation

The NR database species annotation statistics revealed that annotations were assigned to 10 *Bacillus*-related species. Among these, *Paenibacillus* species accounted for 50.68% of the total annotations; *P*. *polymyxa* accounted for 45.31%; *P*. *polymyxa* SQR-21 accounted for only 2.86%; and the remaining seven species collectively accounted for less than 2%. The NR annotation results further confirmed that strain DS-S6 belongs to *P*. *polymyxa*. The NR annotation results not only enhanced data accuracy but also facilitated a deeper understanding of the gene functions of strain DS-S6 and its characteristics within species distribution (Figure 7B).

#### COG functional annotation

The COG annotation clustering identified 24 distinct functional categories. Among these, the pathway with the highest number of annotated functional genes was carbohydrate transport and metabolism, accounting for 11.4% of the total genes. The transcription pathway represented 10.1% of the COG-predicted genes, while amino acid transport and metabolism constituted 9.47%. Furthermore, 107 proteins were involved in secondary metabolites biosynthesis, transport, and catabolism, and 161 proteins were associated with defense mechanisms. This suggests these genes may contribute to the production of antimicrobial substances. Additionally, strain DS-S6 possesses 59 proteins participating in cell motility and 249 proteins involved in cell wall/membrane/envelope biogenesis. These proteins are linked to colonization capability, indicating that this strain exhibits strong colonization ability (Figure 7C).

#### GO functional annotation

A total of 2,850 CDS in the genome of *P*. *polymyxa* DS-S6 were annotated to GO functions, accounting for 54.06% of the total CDS. Among these, 1,849 genes were associated with molecular function, primarily involved in ATP binding, DNA binding, and hydrolase activity. A total of 681 genes were involved in biological processes, with the highest number of genes participating in transmembrane transport (1.56%). Additionally, 1,372 genes were related to cellular components, among which membrane components had the highest number of annotations across all GO functions (Figure 7D). Furthermore, the genome contains genes associated with induced resistance, such as superoxide dismutase activity and peroxidase activity, as well as genes related to pathogen cell wall hydrolysis, including cellulase activity and glucanase activity. It also harbors plant growth-promoting genes, such as those involved in auxin synthesis. These results indicate that the strain has the potential to inhibit pathogens, induce plant resistance, and promote plant growth.

#### KEGG pathway functional annotation

In KEGG pathway analysis 2,945 genes were annotated, encompassing cellular processes, environmental information processing, genetic information processing, metabolism, and organismal systems. All annotated genes participate in 23 metabolic pathways, with 16 pathways containing over 50 genes each (Figure 7E). Within the KEGG level-1 pathways, metabolism contained the most genes (1,954 genes, accounting for 66.04% of the total), followed by environmental information processing (436 genes, 14.80%). KEGG enrichment analysis revealed that the top three most significant pathways were global and overview maps, carbohydrate metabolism, and membrane transport, with 767, 294, and 255 annotated genes, respectively. Additionally, besides the high number of annotated membrane transport genes, strain DS-S6 was also annotated with 71 genes related to cell motility. It is speculated that these genes may be associated with the colonization ability of strain DS-S6.^15^

#### Carbohydrate-active enzymes

In the CAZy database, 270 genes were identified and classified into six families: 140 into glycoside hydrolase (GH) families, 53 into glycosyl transferase (GT) families, 32 into carbohydrate esterase (CE) families, 31 into carbohydrate-binding module families, 12 into polysaccharide lyase families, and two into auxiliary activity families (Figure 7F). The strain has a high abundance of annotated genes for GTs, GHs, and CEs, indicating that it exhibits vigorous growth, active metabolism, and strong reproductive capacity. Studies have shown that hydrolases can degrade fungal cell walls, thereby inhibiting fungal growth.^16^^,^^17^ This strain is capable of producing hydrolases, which is hypothesized to be one of the mechanisms by which it inhibits the growth of plant pathogenic fungi.

### Secondary metabolite synthesis gene cluster

Whole-genome analysis of strain DS-S6 revealed the presence of 18 secondary metabolite biosynthetic gene clusters (BGCs), categorized into seven major types based on their biosynthetic machinery: four nonribosomal peptide synthetase (NRPS) clusters (cluster 1, 10, 11, 16, 17), three *trans*-AT polyketide synthase (PKS)/NRPS hybrid clusters (cluster 3, 5, 15), two ribosomally synthesized and post-translationally modified peptide (RiPP) clusters (cluster 4, 6), three cyclic-lactone-autoinducer clusters (cluster 7, 13, 14), one beta-lactone cluster (cluster 12), three lanthipeptide clusters (cluster 2, 8, 18), and one NRPS-like/cyclic-lactone-autoinducer hybrid cluster (cluster 9). Among these, five BGCs (cluster 1, 2, 8, 12, 17) exhibited 100% similarity to known gene clusters responsible for the biosynthesis of the cyclic lipopeptides fusaricidin family, paenibacillin, paenilan, nostamide A, and polymyxin, respectively. One cluster (cluster 10) exhibited 100% similarity to the gene cluster for the linear lipopeptide tridecaptin. Lower similarity levels were observed for other clusters: cluster 6 showed 40% similarity to the paeninodin (cyclic lipopeptide) BGC, cluster 15 showed 32% similarity to the aurantinin (cyclic peptide) BGC, and cluster 18 showed 28% similarity to an oligosaccharide BGC (Table 3). Significantly, nine gene clusters could not be matched to any characterized BGCs in databases. This strongly suggests that strain DS-S6 represents a promising potential source for the discovery of novel antibiotics and bioactive compounds. These results collectively indicate that strain DS-S6 possesses the genetic capacity to produce secondary metabolites with antimicrobial activity, highlighting its considerable potential for biocontrol applications.

**Table 3:** Secondary metabolites synthetic genetic cluster of strain DS-S6

Cluster ID	Type	Start	End	Similar cluster	Similarity (%)	MIBiG accession
Cluster 1	NRPS	68,297	130,893	Fusaricidin	100	BGC0001152
Cluster 2	Lanthipeptide	991,171	1,016,820	Paenibacillin	100	BGC0000540
Cluster 3	TransAT-PKS,NRPS	1,033,643	1,109,820	–	–	–
Cluster 4	NRPS	1,221,102	1,241,338	–	–	–
Cluster 5	TransAT-PKS,NRPS	1,270,325	1,369,029	–	–	–
Cluster 6	Lassopeptide	1,405,137	1,429,204	Paeninodin	40	BGC0001356
Cluster 7	Cyclic-lactone-autoinducer	1,721,845	1,740,066	–	–	–
Cluster 8	lanthipeptide	1,761,464	1,786,433	Paenilan	100	BGC0001727
Cluster 9	NRPS-like,cyclic-lactone-autoinducer	2.064,250	2,125,446	–	–	–
Cluster 10	NRPS	2,471,747	2,565,248	Tridecaptin	100	BGC0000449
Cluster 11	NRPS	2,722,190	2,800,751	–	–	–
Cluster 12	Beta-lactone	2,951,679	2,982,613	Nostamide A	100	BGC0001479
Cluster 13	Cyclic-lactone-autoinducer	3,026,531	3,043,369	–	–	–
Cluster 14	Cyclic-lactone-autoinducer	3,133,864	3,154,010	–	–	–
Cluster 15	TransAT-PKS, NRPS	3,603,517	3,705,458	Aurantinin	32	BGC0001520
Cluster 16	NRPS	4,439,433	4,464,820	–	–	–
Cluster 17	NRPS	4,974,822	5,054,367	Polymyxin	100	BGC0000408
Cluster 18	Lanthipeptide	5,186,625	5,210,813	S-layer glycan	28	BGC0000796

Note, no similar gene cluster is predicted.

### Functional gene mining

To further understand the growth-promoting mechanisms of the strain, whole-genome sequencing was performed to identify genes associated with plant growth promotion. As shown in Table 4, a total of 79 functional genes were identified, including 26 genes involved in phosphorus solubilization, ninegenes associated with siderophore synthesis and transport, 14 genes related to biofilm formation, and 17 genes involved in nitrogen metabolism. Notably, while the genome contains a complete set of genes for tryptophan biosynthesis—a key precursor for IAA—canonical genes directly responsible for IAA biosynthesis (e.g., *ipdC*, *iaaM*) were not found. Genomic analysis revealed that strain DS-S6 possesses a complete set of genes for tryptophan biosynthesis, which serves as the primary precursor for IAA production in many bacteria. However, canonical genes encoding key enzymes directly involved in converting tryptophan to IAA (e.g., *ipdC* or *iaaM*) were not identified. The detection of IAA by the Salkowski assay suggests that DS-S6 is capable of IAA production, but the underlying biosynthetic pathway remains to be elucidated.

**Table 4:** Predicted genes associated with PGP in DS-S6 genome

Function	Gene	Product
Tryptophan biosynthesis	*trp*A	Tryptophan synthase alpha chain
(IAA precursor)	*trp*B	Tryptophan synthase beta chain
–	*trp*C	Indole-3-glycerol phosphate synthase
–	*trp*D	Anthranilate phosphoribosyltransferase
–	*trp*E	Anthranilate synthase
–	*trp*F	Phosphoribosylanthranilate isomer
–	*aro*G	3-Deoxy-D-arabino-heptulosonate-7-phosphate synthase
–	*aro*A	3-Phosphoshikimate 1-carboxyvinyltransferase
–	*suc*A	2-Oxoglutarate dehydrogenase
–	*ato*A	Acetoacetyl-CoA transferase
–	*ato*B	Acetoacetyl-CoA thiolase
–	*ami*E	Amidase family
–	*aof*H	Acetylpyruvate-CoA transferase
Phosphate solubilization	*phn*X	Phosphonoacetaldehyde hydrolase
–	*phn*Y	2-Aminoethylphosphonate-pyruvate transaminase
–	*phn*P	Phosphoribosyl 1,2-cyclic phosphate phosphodiesterase
–	*pho*A	Alkaline phosphatase
–	*pho*B	Alkaline phosphatase
–	*gdh*A	Glutamate dehydrogenase
–	*phn*C	Phosphonate transport system ATP-binding protein
–	*phn*D	Phosphonate transport system substrate-binding protein
–	*phn*E	Phosphonate transport system permease protein
–	*pst*A	Phosphate transport system permease protein
–	*pst*B	Phosphate transport system ATP-binding protein
–	*pst*C	Phosphate transport system permease protein
–	*pst*S	Phosphate transport system substrate-binding protein
–	*ack*A	Acetate kinase
–	*glt*A	Citrate synthase
–	*glt*B	Glutamate synthase (NADPH) large chain
–	*glt*D	Glutamate synthase (NADPH) large chain
–	*aro*A	3-deoxy-7-phosphoheptulonate synthase
–	*aro*B	3-dehydroquinate synthase
–	*aro*C	Chorismate synthase
–	*aro*D	3-dehydroquinate dehydratase
–	*aro*E	Shikimate dehydrogenase
–	*aro*K	Shikimate kinase
–	*ack*A	Acetate kinase
–	*pyk*	Pyruvate kinase
–	*ldh*	L-lactate dehydrogenase
Siderophore production	*dhb*F	Amino acid adenylation domain-containing protein
–	*dhb*A	2,3-dihydro-2,3-dihydroxybenzoate dehydrogenase
–	*dhb*B	Isochorismatase
–	*dhb*C	Isochorismate synthase DhbC
–	*dhb*E *tro*A *tro*B *tro*C *tro*D	(2,3-dihydroxybenzoyl) adenylate synthase Iron transport system substrate-binding protein Iron transport system ATP- binding protein Iron transport system permease protein Iron transport system permease protein
Biofilm formation	*cys*E	Serine O-acetyltransferase
–	*trp*E	Anthranilate synthase component
–	*csr*A	Carbon storage regulator
–	*fli*A	RNA polymerase sigma factor FliA
–	*rpo*E	DNA-directed RNA polymerase subunit delta
–	*lux*S	S-ribosylhomocysteine lyase
–	*mot*A	Chemotaxis protein MotA
–	*mot*B *flg*M	Chemotaxis protein MotB Negative regulator of flagellin synthesis FlgM
–	*kin*B	Two-component system, sporulation sensor kinase B
–	*spo*0A *spo*0F *deg*U	Two-component system, response regulator, stage 0 sporulation protein A Two-component system, response regulator, stage 0 sporulation protein F Two-component system, NarL family, response regulator DegU
–	*deg*S	Two-component system, NarL family, sensor histidine kinas DegS
Nitrate/nitrite reduction	*car*A	Carbamoyl-phosphate synthase small subunit
(nitrogen metabolism)	*car*B	Carbamoyl-phosphate synthase large subunit
–	*pur*F	Amidophosphoribosyltransferase
–	*ser*C *gln*A *glt*B *nar*G *nar*Z *nar*H *nar*Y *nar*B *nas*A *nar*K *nir*A *nir*B *nir*D	Phosphoserine aminotransferase Glutamine synthetase Glutamate synthase (NADPH) large chain Nitrate reductase alpha subunit Nitrate reductase alpha subunit Nitrate reductase beta subunit Nitrate reductase beta subunit Nitrate reductase beta subunit Assimilatory nitrate reductase catalytic subunit MFS transporter, NNP family, nitrate/nitrite transporter Ferredoxin-nitrite reductase Nitrite reductase (NADH) large subunit Nitrite reductase (NADH) small subunit
–	*cyn*T	Carbonic anhydrase

Among the genes related to phosphorus solubilization, there are inorganic phosphorus solubilization genes such as *gdh*A and *pho*A, organic phosphorus degradation genes such as *phn*X and *phn*Y, and transport/auxiliary genes such as *phn*P and *phn*C. Bacteria generally absorb phosphorus through specific phosphorus compound transport proteins located on the plasma membrane.^18^ These include inorganic phosphate-specific transport proteins encoded by the *pst*S, *pst*C, *pst*A, and *pst*B genes, as well as phosphonate transport proteins encoded by the *phn*E, *phn*D, and *phn*C genes. By searching the prokaryotic genome annotation pipeline annotation results, we found that strain DS-S6 carries multiple functional genes related to organic acid synthesis, including those for acetate (*ack*A), pyruvate (*pyk*), shikimate (*aro*A, *aro*B, *aro*C, *aro*D, *aro*E, and *aro*K), lactate (*ldh*), and citrate (*glt*A). These organic acids enhance soil acidity, promoting the conversion of insoluble phosphates into plant-available free phosphates, thereby enabling the efficient utilization of inorganic phosphorus.

Furthermore, through annotation of the whole genome of strain DS-S6, we identified a complete *dhb* gene cluster (*dhb*ACEBF) encoding the synthesis of the siderophore. Additionally, we discovered genes directly involved in siderophore uptake: *tro*C and *tro*D. These genes are core components of the TonB-ExbB-ExbD system, which provides the necessary energy for the transport of siderophore-iron complexes into the periplasmic space through outer membrane receptors.^19^ The absence of these genes would result in defects in siderophore utilization.

Among the genes related to biofilm formation that were identified, the *cys*E gene participates in amino acid metabolism, providing precursors for biofilm protein synthesis^20^; the *csr*A gene regulates carbon source utilization, indirectly intervening in the energy supply for biofilm formation^21^; the *fli*A gene controls genes associated with bacterial motility, indirectly affecting biofilm development^22^; and the *rpo*E gene maintains the essential cell envelope integrity and homeostasis during biofilm formation.^23^ Additionally, we identified key quorum-sensing genes such as *lux*S, which influences biofilm formation, along with flagellar motility-related genes *mot*A, *mot*B, and *flg*M, as well as genes involved in the biofilm formation pathway of Bacillus, including *kin*B, *spo*0A, *spo*0F, *deg*U, and *deg*S.

Genes such as *car*A, *car*B, *pur*F, and *ser*C are involved in nitrogen metabolism and ammonia assimilation, encoding subunits of carbamoyl phosphate synthase and aminotransferases.^24^ The *nar*G, *nar*Z, *nar*H, *nar*Y, *nar*B, and *nas*A genes encode nitrate reductases that catalyze the reduction of nitrate (NO_3_^−^) to nitrite (NO_2_^-^),^25^ while *nir*B and *nir*D encode NADH-dependent nitrite reductases that further reduce nitrite to ammonia (NH_3_/NH_4_^+^).^26^ These genetic features indicate that strain DS-S6 is capable of assimilatory and dissimilatory nitrate/nitrite reduction, enabling it to utilize inorganic nitrogen sources (e.g., NO_3_^−^ or NO_2_^-^) for growth and potentially enhance nitrogen availability to plants. Notably, no canonical nitrogen fixation genes (e.g., *nif*H, *nif*D, and *nif*K) were identified in the genome, confirming that DS-S6 does not possess the capacity for biological nitrogen fixation (i.e., conversion of atmospheric N_2_ to ammonia).^27^

## Discussion

The fungal cell wall is composed of polysaccharides, with major constituents including chitin, β-1,3-glucan, mannan, cellulose, and galactose polymers, which has a complex structure.^28^ The fungal cell wall plays a crucial protective role in the survival of fungi; disruption of the cell wall structure leads to rupture of the plasma membrane and cell lysis.^29^ Under the action of chitinase, chitin is hydrolyzed to produce N-acetylglucosamine (GlcNAc). Enzymes such as glucanase, cellulase, and peptidoglycan-degrading enzymes can act synergistically with chitinase to disrupt the integrity of the fungal cell wall structure.^30^ This subsequently inhibits the growth of fungal hyphae and the germination of spores, thereby achieving biological control. Therefore, various cell wall-degrading enzymes can also serve as important antifungal substances for the control of fungal diseases. In this study, plate-based assays and enzyme activity measurements confirmed that the strain DS-S6 is capable of producing protease, cellulase, chitinase, and β-1,3-glucanase. Genome analysis of strain DS-S6 identified 26 genes associated with the four hydrolytic enzymes mentioned above: 12 genes related to chitinase, three genes related to cellulase, six genes related to protease, and five genes related to glucanase (Table S1).

The key to *P*. *polymyxa* exhibiting biocontrol potential lies in its ability to produce antimicrobial secondary metabolites.^12^ Genomic analysis of *P*. *polymyxa* DS-S6 revealed 18 secondary metabolite biosynthetic gene clusters, primarily composed of NRPS and transAT-PKS systems. These NRPS and transAT-PKS are responsible for synthesizing the two major classes of secondary metabolites in *P*. *polymyxa*, which play significant roles in green disease control, pharmaceutical development, and pesticide production.^31^ Polymyxin‌ is a class of polycationic cyclic peptide antibiotics, including components A, B, C, and D, exhibiting broad-spectrum antibacterial activity, particularly strong inhibition against gram-negative bacteria.^32^ Fusaricidin‌ is a cyclic lipopeptide compound primarily active against gram-positive bacteria and various pathogenic fungi.^33^ These antibiotics are commonly used for plant disease control, especially against the globally recognized pathogen *Fusarium*, demonstrating excellent control efficacy. Paenilipoheptin is a family of lipoheptapeptide antibiotics produced by *P*. *polymyxa*, characterized by a fatty acyl chain attached to a cyclic heptapeptide core.^34^ The linear cationic lipopeptide ‌tridecaptin‌ inhibits cell wall peptidoglycan synthesis, leading to bacterial lysis, thereby exhibiting antibacterial effects against multiple gram-negative bacteria.^35^

**Figure 8:**
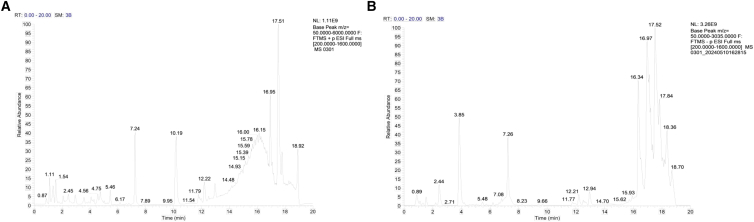
Total ion chromatogram of strain DS-S6 fermentation broth (A) Positive ion mode; (B) negative ion mode.

To identify the antimicrobial compounds produced by strain DS-S6, the metabolites in its fermentation broth were analyzed by LC-MS. Seven lipopeptides were identified in this analysis, with their corresponding molecular masses provided in Figure 8 and Table 5. These included polymyxin B1 (1,202.34 Da) and B2 (1,181.71 Da), the fusaricidin series A-D (882.52, 896.56, 946.54, and 960.53 Da, respectively), and paenibacillin B (1,468.74 Da). The LC-MS experimental results were consistent with the earlier predictions from the secondary metabolic gene cluster analysis. Among the detected lipopeptides, fusaricidins, and paenibacillin are known to exhibit strong antifungal activity against *Fusarium* spp., whereas polymyxins primarily target Gram-negative bacteria and are unlikely to contribute to the observed suppression of *F*. *solani*. This suggests that fusaricidin and paenibacillin-type compounds are the most probable mediators of DS-S6’s biocontrol effect. While our multi-omics approach strongly implicates fusaricidins and paenibacillin in DS-S6’s antifungal activity, future work employing purified compounds or knockout mutants will be required to definitively assign biocontrol function to individual metabolites.

**Table 5:** Lipopeptides produced by DS-S6 and their biological activities

Compound name	Molecular weight	Molecular formula	Retention time (min)	Detection ion mode	Biological activity
Polymyxin B2	1,188.71	C_55_H_96_N_16_O_13_	14.93	Positive	Antibacterial
PolymyxinB1	1,202.34	C_56_H_98_N_16_O_13_	15.15	Positive	Antibacterial
Fusaricidin D	960.53	C_46_H_76_N_10_O_12_	15.59	Positive	Antifungal
Fusaricidin C	946.54	C_45_H_74_N_10_O_12_	15.78	Positive	Antifungal
Fusaricidin B	896.56	C_42_H_76_N_10_O_11_	16.00	Positive	Antifungal
Fusaricidin A	882.52	C_41_H_74_N_10_O_11_	16.15	Positive	Antifungal
PaenibacillinB	1,468.74	C_67_H_113_N_15_O_17_	18.92	Positive	Antifungal

While our multi-omics approach provides compelling evidence for the role of fusaricidin and paenibacillin in suppressing *Fusarium* root rot, it is crucial to acknowledge that biocontrol efficacy is highly strain-dependent, even within the same species such as *P*. *polymyxa*.^36^ The genetic repertoire, metabolite profile, and host interaction dynamics can vary dramatically among isolates, limiting the generalizability of findings from a single strain. Moreover, alternative mechanisms—including induced systemic resistance, siderophore-mediated iron competition, or niche exclusion through robust rhizosphere colonization—may also contribute synergistically to disease suppression. Future studies employing mutant strains deficient in specific lipopeptides will be essential to disentangle the relative contributions of direct antimicrobial action versus indirect plant-mediated defenses.

Notably, our LC-MS analysis also detected polymyxin, a lipopeptide with potent activity against Gram-negative bacteria and clinical relevance as a last-resort antibiotic. Although polymyxin does not contribute to antifungal activity, its production by environmental *P*. *polymyxa* strains raises potential biosafety and ecological concerns. As highlighted in a recent review on endophyte-based sustainable agriculture, the environmental release of microbes carrying antibiotic biosynthesis genes warrants careful risk assessment to prevent unintended consequences such as horizontal gene transfer or disruption of native soil microbiomes.^36^ While DS-S6 shows promise as a bioinoculant, responsible deployment requires thorough evaluation of its genomic stability, horizontal transfer potential, and non-target effects prior to field application.

**Figure 9:**
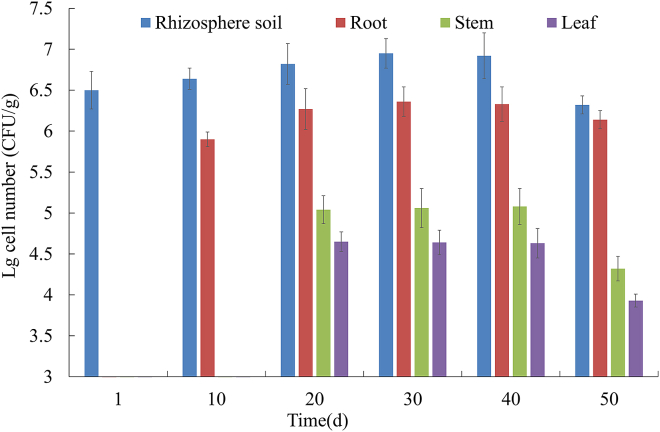
Colonization of DS-S6-GFP in rhizospheric soil and root, stem and leaf tissues

Studies have shown that the biocontrol capacity of biocontrol bacteria is related to their ability to form biofilms.^37^^,^^38^ To better compete for nutrients within plants, biocontrol bacteria first need to stably survive in the plant, and biofilm formation determines their colonization ability within the plant.^39^ To elucidate the colonization patterns of strain DS-S6 in the rhizosphere soil of *S*. *miltiorrhiza* and its root, stem, and leaf tissues, we conducted colonization experiments using the GFP-labeled strain DS-S6 according to the method reported by Sa et al.^40^ The constructed strain DS-S6-GFP was applied to the roots of *S*. *miltiorrhiza* seedlings, and samples were collected at 1, 10, 20, 30, 40, and 50 days post-inoculation to detect the colonization of strain DS-S6-GFP. As shown in Figure 9, DS-S6-GFP successfully colonized the rhizosphere and internal tissues, with populations persisting for at least 50 days—particularly on root surfaces, the primary site of *Fusarium* infection. Although GFP-tagged DS-S6 successfully colonized the rhizosphere and plant tissues of *S*. *miltiorrhiza*, we acknowledge that the current study did not directly correlate colonization density with biocontrol efficacy or growth promotion. Future work combining quantitative tracking (e.g., qPCR) with phenotypic assays will be needed to establish such a dose-response relationship. Nevertheless, the persistent presence of DS-S6 in the rhizosphere—particularly at the root surface where *Fusarium* infection initiates—provides a plausible spatial basis for its observed antifungal activity.

Although IAA production by *P*. *polymyxa* DS-S6 was detected using the Salkowski colorimetric assay, it should be noted that this method has certain limitations. The Salkowski reagent can react non-specifically with various indole-containing compounds, potentially leading to false-positive results. Moreover, the assay provides only semi-quantitative data and may not accurately reflect the true IAA concentration. Nevertheless, due to its simplicity and low cost, the Salkowski assay remains a widely used preliminary screening tool for plant growth-promoting rhizobacteria.^41^ To conclusively confirm IAA production and elucidate its biosynthetic pathway, future work will employ high-specificity analytical techniques such as HPLC or LC-MS/MS, combined with gene expression analysis of putative IAA-related genes. More broadly, several plant growth-promoting traits described herein—including phosphate solubilization, siderophore-mediated iron acquisition, and phytohormone modulation—are primarily inferred from genomic annotations. Although these predictions are biologically plausible, they require functional validation through targeted experiments such as gene knockout or complementation, transcriptomic profiling under plant-mimicking conditions, and isotopic labeling assays. Future work integrating multi-omics approaches with in planta phenotyping will be essential to move beyond genetic potential and establish causal links between genotype and plant-beneficial phenotype.

Collectively, our integrated genomic, metabolomic, and phenotypic characterization positions DS-S6 as a promising candidate for sustainable management of *Fusarium* root rot in *S*. *miltiorrhiza*. However, as emphasized by Kumar et al., the transition from laboratory discovery to field-ready bioinoculants demands rigorous validation of host compatibility, environmental resilience, and ecological safety.^36^ Future work should prioritize multi-omics-guided strain optimization, dose-response relationships between colonization and protection, and field trials under diverse agroecological conditions to realize the full potential of endophytic probiotics in climate-resilient agriculture. In this study, we integrated genomic, metabolomic, and phenotypic approaches to characterize the endophytic strain *P*. *polymyxa* DS-S6 isolated from *S*. *miltiorrhiza*. The complete genome sequence revealed a rich genetic repertoire potentially associated with plant growth promotion and biocontrol activities, including phosphate solubilization, siderophore production, IAA-like compound secretion, and synthesis of antifungal lipopeptides. LC-MS analysis confirmed the production of fusaricidin and paenibacillin, which are likely contributors to the observed suppression of *F*. *solani*-induced root rot in pot experiments. GFP-labeling further demonstrated that DS-S6 can stably colonize the rhizosphere and internal tissues of *S*. *miltiorrhiza* for at least 50 days, providing a plausible basis for its biocontrol effect.

While these findings support the potential of DS-S6 as a biocontrol agent against *Fusarium* root rot in *S*. *miltiorrhiza*, it should be noted that most functional annotations are based on genomic predictions and require experimental validation. Moreover, efficacy has only been evaluated under controlled greenhouse conditions; field trials and safety assessments—including ecological implications of polymyxin production—are necessary before practical application. Nevertheless, DS-S6 represents a promising candidate for further development as a microbial inoculant for *S*. *miltiorrhiza*.

### Limitations of the study

Most functional annotations in this study are based on genomic predictions and require experimental validation through gene knockout or complementation studies. Moreover, biocontrol efficacy was evaluated only under controlled greenhouse conditions, and field trials under diverse agroecological conditions together with ecological safety assessments are necessary before practical application.

## Resource availability

### Lead contact

Requests for further information and resources should be directed to and will be fulfilled by the lead contact, Meimei Song (mmsong.shuxue@163.com).

### Materials availability

*P*. *polymyxa* DS-S6 is available from the lead contact upon request.

### Data and code availability

• The genome sequence of P. polymyxa DS-S6 has been deposited at NCBI under BioProject ID PRJNA1301861. The accession number is listed in the key resources table.
• This study does not report original code.
• Any additional information required to reanalyze the data reported in this study is available from the lead contact upon request.

## Acknowledgments

This work was financially supported by the Shandong Traditional Chinese Medicine Science and Technology Development Plan Project (2019–0347). We thank Beijing Allwegene Technology Co., Ltd. for whole-genome sequencing services. We are grateful to the members of the School of Life Sciences, Shandong First Medical University, for their technical assistance.

## Author contributions

R.S.: participated in experiment implementation, data collection and analysis, and manuscript writing and revision; Z.C. and Q.L.: participated in experiment implementation and data analysis, and assisted in manuscript revision; X.L., S.M., and G.Z.: participated in experiment implementation; J.Z.: supervised experiment implementation, reviewed experimental processes and data, and provided revision suggestions; Y.Z.: supervised experiment implementation and reviewed experimental processes and data; M.S.: responsible for research conception, experimental design and planning, guided research direction and data interpretation, and reviewed study writing and finalization. All authors have read and agreed to the published version of the manuscript.

## Declaration of interests

The authors declare no competing interests.

## STAR★Methods

### Key resources table

**Table:** 

REAGENT or RESOURCE	SOURCE	IDENTIFIER
**Bacterial and virus strains**		

*Paenibacillus polymyxa* DS-S6	This study	PRJNA1301861
*Escherichia coli* DH5α	Laboratory stock	N/A

**Experimental models: Organisms/strains**		

*Fusarium solani*	Sa et al., 2022^4^	N/A
*Fusarium oxysporum*	Sa et al., 2022^4^	N/A
*Rhizoctonia solani*	Sa et al., 2022^4^	N/A
*Alternaria alternata*	Sa et al., 2022^4^	N/A

**Chemicals, peptides, and recombinant proteins**		

Luria-Bertani (LB) broth	Solarbio	L1010
LB agar	Solarbio	L1015
Potato dextrose agar (PDA)	BD Difco	213400
Carbendazim	Sigma-Aldrich	378674
L-tryptophan	Sigma-Aldrich	T0254
Chrome Azurol S (CAS)	Sigma-Aldrich	227080
Congo red	Sigma-Aldrich	C6767
Glutaraldehyde	Sigma-Aldrich	G5882
Osmium tetroxide	Sigma-Aldrich	75632
Chloramphenicol	Sigma-Aldrich	C0378

**Critical commercial assays**		

Bacterial DNA Kit	Omega Bio-Tek	D3350

**Deposited data**		

*P. polymyxa* DS-S6 genome	NCBI	PRJNA1301861

**Oligonucleotides**		

16 S rDNA forward: 27 F (5′-AGAGTTTGATCCTGGCTCAG-3′)	This study	N/A
16 S rDNA reverse: 1492 R (5′-GGTTACCTTGTTACGACTT-3′)	This study	N/A

**Recombinant DNA**		

Plasmid pHT01-P43GFPmut3a	Sa et al., 2021^40^	N/A

**Software and algorithms**		

MEGA 11.0	Tamura et al., 2021	https://www.megasoftware.net/
antiSMASH 5.2.0	Blin et al., 2021	https://antismash.secondarymetabolites.org/
SPSS 26.0	IBM Corp.	https://www.ibm.com/spss
Glimmer	Delcher et al., 1999	https://ccb.jhu.edu/software/glimmer/
GeneMarkS	Besemer et al., 2001	https://topaz.gatech.edu/GeneMark/
Prodigal	Hyatt et al., 2010	https://github.com/hyattpd/Prodigal
Hmmscan	Eddy, 2011	http://hmmer.org/

**Other**		

Scanning electron microscope	Hitachi	SU8010
Fluorescence microscope	Olympus	BX53

### Experimental model and study participant details

P. polymyxa DS-S6 was isolated from Salvia miltiorrhiza stems. F. solani, F. oxysporum, R. solani, and A. alternata were maintained on potato dextrose agar (PDA) at 28 ± 2°C. S. miltiorrhiza seedlings (three months old) were grown in a greenhouse under natural light conditions (16 h light/8 h dark cycle). This study did not involve animals, human participants, or cell lines. Therefore, sex, gender, authentication, and mycoplasma testing are not applicable.

### Method details

Detailed methods are provided in the Materials and Methods section. Key procedures include antifungal assays, PGP trait tests, pot experiments, genome sequencing (NCBI PRJNA1301861), and LC-MS analysis.

### Quantification and statistical analysis

Data are mean ± SD. Student's t-test for two groups; one-way ANOVA with LSD for multiple comparisons. Significance: ∗p∗ < 0.05. SPSS 26.0 was used. SPSS 26.0 (https://www.ibm.com/spss) was used.
