# Supplementary material for: Integrated genomic and phenotypic analysis of an endophytic bacterium reveals biocontrol and plant growth-promoting mechanisms
Source: iScience. 2026 Jun 14;29(6):116210. doi: 10.1016/j.isci.2026.116210 (PMC13267615; doi:10.1016/j.isci.2026.116210)
Supplement: Document S1. Figures S1–S3 and Table S1 [file mmc1.pdf]

iScience, Volume *29*

Supplemental information

Integrated genomic and phenotypic analysis of an endophytic bacterium reveals biocontrol and plant growth-promoting mechanisms

Rongbo Sa, Zhijie Cao, Qin Luo, Xinru Liu, Shuxin Ma, Guoqing Zhang, Junli Zhang, Yachao Zhang, and Meimei Song

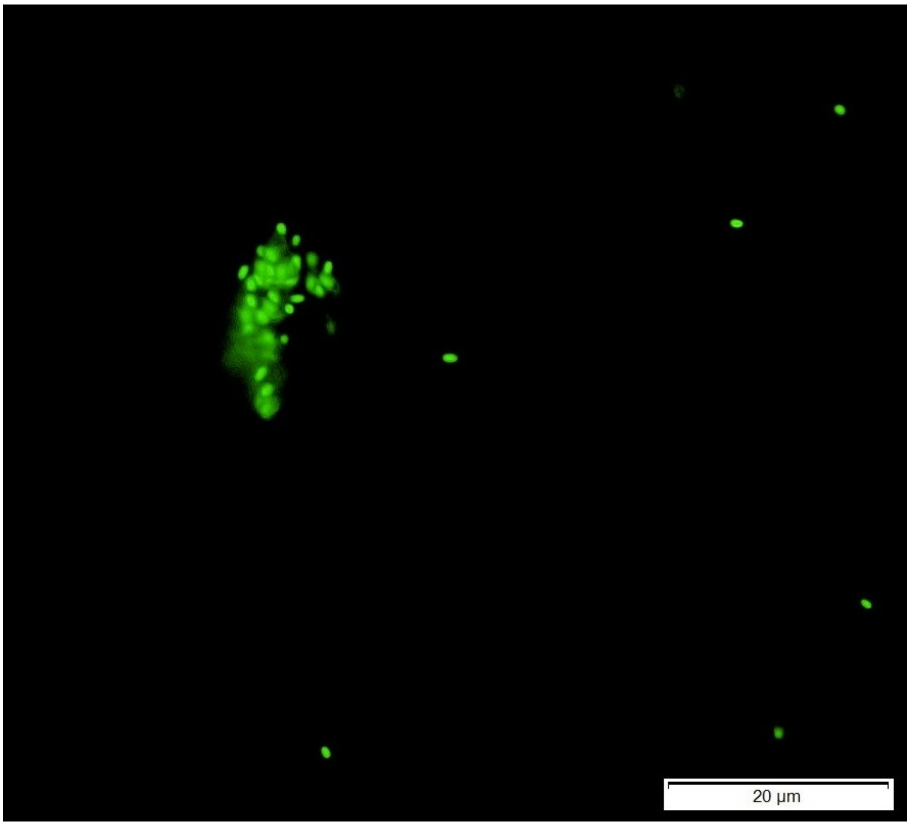

Figure S1. Fluorescence of GFP-labeled DS-S6-GFP strain. Scale bar = 20 μm.

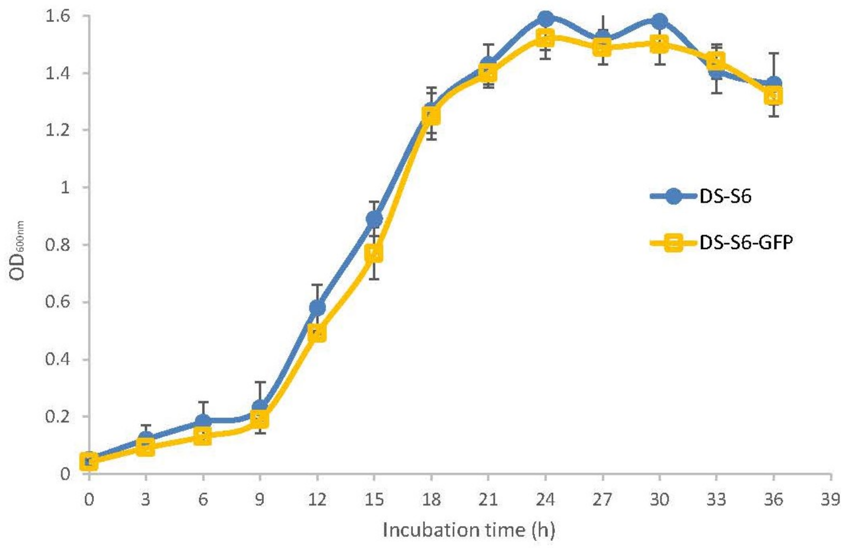

Figure S2. Growth kinetics of wild-type DS-S6 and DS-S6-GFP in LB broth over 36 h. Data are means ± SD (n = 3).

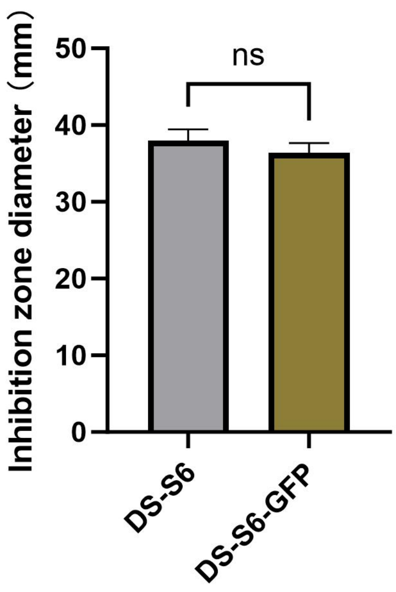

Figure S3. Comparison of antifungal activity against *F. solani* between wild-type DS-S6 and DS-S6-GFP.

Table S1. Genes associated with hydrolytic enzymes (chitinase, cellulase, protease, and glucanase) in the genome of *P. polymyxa* DS-S6.

Function	Gene	Product
Chitinas synthesis	*chi*A	Chitinase A
	*nag*A	N-acetylglucosamine-6-phosphate deacetylase
	*nag*B	Glucosamine-6-phosphate deaminase
	*chi*E	Putative chitobiose transport system substrate-binding protein
	*chi*F	Putative chitobiose transport system permease protein
	*chi*G	Putative chitobiose transport system permease protein
	*sec*A	Preprotein translocase subunit
	*sec*E	Preprotein translocase subunit
	*sec*G	Preprotein translocase subunit
	*sec*Y	Preprotein translocase subunit
	*tat*A	Sec-independent protein translocase protein
	*tat*C	Sec-independent protein translocase protein
Cellulase synthesis	*cel*A	Endo-β-1,4-glucanase
	*cel*B	Exo-β-1,4-glucanase
	*cel*C	β-glucosidase
Protease synthesis	*prt*C	Collagenase
	*ctp*A	Carboxyl-terminal protease
	Lon	ATP-dependent Lon protease
	*deg*P	Serine protease
	*deg*S	Two-component system, NarL family, sensor histidine kinase
	*deg*U	Two-component system, NarL family, response regulator
Glucanase synthesis	*bgl*A	6-phospho-β-glucosidase
	*bgl*B	β-glucosidase
	*bgl*X	β-glucosidase
	*bgl*F	β-glucoside PTS system EIICBA component
	*bgl*P	β-glucoside PTS system EIICBA component
